# Comparative Effectiveness of GLP‐1RAs, SGLT2is and DPP4is for Cardiovascular Outcomes in Type 2 Diabetes: An Empirically Calibrated Target Trial Emulation Study

**DOI:** 10.1111/dom.71244

**Published:** 2026-08-27

**Authors:** Bingyu Zhang, Ting Zhou, Huilin Tang, Yiwen Lu, Dazheng Zhang, Jiajie Chen, Tianchu Lyu, Kai Zheng, David A. Asch, Yong Chen

**Affiliations:** ^1^ Center for Health AI and Synthesis of Evidence (CHASE) University of Pennsylvania Philadelphia Pennsylvania USA; ^2^ The Graduate Group in Applied Mathematics and Computational Science, School of Arts and Sciences University of Pennsylvania Philadelphia Pennsylvania USA; ^3^ Department of Biostatistics, Epidemiology, and Informatics University of Pennsylvania Perelman School of Medicine Philadelphia Pennsylvania USA; ^4^ Institute for Clinical and Translational Science University of California, Irvine Irvine California USA; ^5^ Department of Informatics, Donald Bren School of Information and Computer Sciences University of California, Irvine Irvine California USA; ^6^ Department of Medicine, Perelman School of Medicine University of Pennsylvania Philadelphia Pennsylvania USA; ^7^ Penn Center for Health Incentives and Behavioral Economics University of Pennsylvania Philadelphia Pennsylvania USA; ^8^ Wharton School University of Pennsylvania Philadelphia Pennsylvania USA; ^9^ Leonard Davis Institute of Health Economics, University of Pennsylvania Philadelphia Pennsylvania USA; ^10^ Division of General Internal Medicine University of Pennsylvania Philadelphia Pennsylvania USA; ^11^ Penn Medicine Center for Evidence‐Based Practice (CEP) Philadelphia Pennsylvania USA; ^12^ Penn Institute for Biomedical Informatics (IBI) Philadelphia Pennsylvania USA

## 1 Background

Randomised controlled trials have demonstrated cardiovascular benefits of glucagon‐like peptide‐1 receptor agonists (GLP‐1RAs) and sodium‐glucose cotransporter‐2 inhibitors (SGLT2is) [1, 2], but head‐to‐head comparisons between these agents and dipeptidyl peptidase‐4 inhibitors (DPP4is) are lacking. Observational studies have provided valuable comparative evidence, but many have focused on populations with established cardiovascular disease, selected treatment comparisons or noncardiovascular primary outcomes [3, 4, 5]. As a result, uncertainty remains regarding comparative cardiovascular effectiveness of GLP‐1RAs, SGLT2is and DPP4is across a broader real‐world population with Type 2 diabetes (T2D). Moreover, as with observational studies, residual systematic error may persist despite careful adjustment for measured confounding. Therefore, we compared the cardiovascular effectiveness of GLP‐1RAs, SGLT2is and DPP4is in patients with T2D using an empirically calibrated target trial emulation (TTE) framework with negative control outcome (NCO) calibration.

## 2 Methods

This TTE study (Table S1) used electronic health records from five hospitals and affiliated outpatient clinics within Penn Medicine from January 2018 through September 2024. The cohort entry window spanned from January 2019 through September 2024, with 2018 serving as the baseline period to assess prior healthcare engagement and measure baseline covariates. We emulated new‐user, active‐comparator trials among adults aged 18 years or older with T2D who newly initiated one of GLP‐1RAs, SGLT2is or DPP4is. The date of the first eligible prescription is defined as time zero (index date). Patients who initiated more than one study drug class on the index date were excluded. To align eligibility assessment with treatment initiation and avoid immortal time bias, all baseline covariates, exclusions and treatment assignment were defined at or before this index date.

Eligible patients were required to have at least 1 health care encounter in the preceding year to ensure adequate baseline information. We also excluded patients with recorded use of any GLP‐1RA, SGLT2i or DPP4i, Type 1 diabetes, end‐stage renal disease or a recorded diagnosis of any study cardiovascular outcome during the 1‐year baseline period (Figure S1). We conducted three separate pairwise comparison TTEs: GLP‐1RAs versus DPP4is, SGLT2is versus DPP4is and GLP‐1RAs versus SGLT2is.

Outcomes included nonfatal myocardial infarction (MI), nonfatal stroke, cardiovascular death, hospitalisation for unstable angina (UA) and 3‐point and 4‐point major adverse cardiovascular events (MACE), defined respectively as the composite of nonfatal MI, nonfatal stroke and cardiovascular death and the composite of those events plus hospitalisation for UA. We estimated the intention‐to‐treat effect of treatment initiation, with patients analysed according to their study drug class at the index date, regardless of subsequent treatment changes or discontinuation. Follow‐up began at index date and ended at the earliest of onset of outcome, death, 1 year after the last recorded encounter or study end.

Baseline covariates were selected a priori based on previous literature and clinical and epidemiological knowledge and included variables plausibly associated with both treatment selection and cardiovascular outcomes [6]. These included demographics, body mass index, systolic and diastolic blood pressure, insurance type, zip code‐level median household income, healthcare utilisation, Charlson Comorbidity Index (CCI), more than 100 pre‐existing comorbidities, baseline medication use and T2D complications (Table S2). Missing race or ethnicity information was included in the ‘Other/Unknown’ category. Missing values for body mass index and blood pressure, which accounted for approximately 4%–8% of observations, were imputed using the corresponding group mean. For each pairwise comparison, patients were matched 1:1 using nearest‐neighbour matching without replacement on LASSO‐estimated large‐scale propensity scores (PS), with a calliper width of 0.2 standard deviations of the logit of PS. Covariate balance was assessed using standardised mean differences, with values less than 0.1 indicating adequate balance (Figure S2).

For each outcome, we estimated incidence rate ratios (IRRs) and 95% confidence intervals (CIs) using modified Poisson regression, including follow‐up time as an offset to account for differential person‐time after matching. To evaluate and reduce residual systematic bias, we further applied NCO calibration as the primary debiasing procedure. Prespecified NCOs, selected from prior literature and expert review [3], were outcomes not plausibly affected by the study drugs but expected to share a similar confounding structure as the cardiovascular outcomes (Table S3). We analysed each NCO using the same cohort construction, matching strategy, follow‐up and outcome model as used for the primary analyses. The resulting distribution of log‐effect estimates was used to estimate an empirical null distribution of residual systematic bias (Figure S3). Primary outcome estimates were then calibrated against this empirical null to obtain bias‐adjusted effect estimates and CIs. Additional methodological details are provided in the Supplement and prior methodological references [7, 8, 9]. Descriptive all‐cause mortality results, sensitivity analyses using the per‐protocol approach, excluding tirzepatide from the GLP‐1RA group and reporting 3‐year risk differences and exploratory subgroup analyses by age, sex and race and ethnicity, are reported in the Tables S7 and S8 and Figures S4–S15. In the per‐protocol analysis, follow‐up was additionally censored at discontinuation of the index treatment or initiation of another study drug class.

The University of Pennsylvania institutional review board approved the study with a waiver of informed consent. This report follows the STROBE reporting guideline. All analyses were performed using R version 4.4.0. Statistical significance was set at a *p*‐value < 0.05 (two‐tailed).

## 3 Results

A total of 30 040 individuals (mean [SD] age 62.3 [13.1] years; 51.4% male; 50.2% Non‐Hispanic White, 31.3% Non‐Hispanic Black) were included (Table 1). The mean follow‐up was 2.7 years. After 1:1 propensity‐score matching, the GLP‐1RA versus SGLT2i comparison included 7223 patients per group, the GLP‐1RA versus DPP4i comparison included 5934 per group and the SGLT2i versus DPP4i comparison included 7505 per group. Outcome event counts, total follow‐up time and crude incidence rates for all six cardiovascular outcomes across the three pairwise treatment comparisons are reported in Tables S7 and S8. Before matching, treatment groups differed across several clinical characteristics; after large‐scale propensity score matching, baseline covariates were well balanced in all pairwise comparisons, with standardised mean differences below 0.1 for all matched variables (Figure S2 and Tables S4–S6).

**TABLE 1:** Patient baseline characteristics before matching.

	GLP‐1RA (*N* = 10 174)	SGLT2i (*N* = 11 249)	DPP4i (*N* = 8617)	Overall (*N* = 30 040)
Age				
Years; mean (SD)	57.4 (12.7)	63.9 (12.1)	66.1 (13.1)	62.3 (13.1)
Sex				
Female	5833 (57.3%)	4503 (40.0%)	4271 (49.6%)	14 607 (48.6%)
Male	4341 (42.7%)	6746 (60.0%)	4346 (50.4%)	15 433 (51.4%)
Race and ethnicity				
Non‐Hispanic White	4622 (45.4%)	5875 (52.2%)	4572 (53.1%)	15 069 (50.2%)
Non‐Hispanic Black	4053 (39.8%)	3177 (28.2%)	2174 (25.2%)	9404 (31.3%)
Hispanic	523 (5.1%)	593 (5.3%)	503 (5.8%)	1619 (5.4%)
Asian	333 (3.3%)	631 (5.6%)	658 (7.6%)	1622 (5.4%)
Other/Unknown	643 (6.3%)	973 (8.6%)	710 (8.2%)	2326 (7.7%)
CCI score				
< 3	3351 (32.9%)	2882 (25.6%)	3004 (34.9%)	9237 (30.7%)
≥ 3	6823 (67.1%)	8367 (74.4%)	5613 (65.1%)	20 803 (69.3%)
Insurance				
Commercial	4688 (46.1%)	4036 (35.9%)	2613 (30.3%)	11 337 (37.7%)
Medicaid	1509 (14.8%)	957 (8.5%)	676 (7.8%)	3142 (10.5%)
Medicare	3039 (29.9%)	4411 (39.2%)	4304 (49.9%)	11 754 (39.1%)
Self‐Pay/Other	938 (9.2%)	1845 (16.4%)	1024 (11.9%)	3807 (12.7%)
Zip code‐level median household income				
< $50 000	3131 (30.8%)	2431 (21.6%)	1527 (17.7%)	7089 (23.6%)
$50 000–$100 000	4675 (46.0%)	5595 (49.7%)	5032 (58.4%)	15 302 (50.9%)
≥ $100 000	2368 (23.3%)	3223 (28.7%)	2058 (23.9%)	7649 (25.5%)
Number of inpatient visits				
0	9071 (89.2%)	9671 (86.0%)	6931 (80.4%)	25 673 (85.5%)
≥ 1	1103 (10.8%)	1578 (14.0%)	1686 (19.6%)	4367 (14.5%)
Number of outpatient visits				
< 10	2477 (24.3%)	3543 (31.5%)	3052 (35.4%)	9072 (30.2%)
≥ 10	7697 (75.7%)	7706 (68.5%)	5565 (64.6%)	20 968 (69.8%)
Number of ED visits				
0	8845 (86.9%)	10 133 (90.1%)	7694 (89.3%)	26 672 (88.8%)
≥ 1	1329 (13.1%)	1116 (9.9%)	923 (10.7%)	3368 (11.2%)
Year of cohort entry				
2019	2219 (21.8%)	1264 (11.2%)	2747 (31.9%)	6230 (20.7%)
2020	1438 (14.1%)	1217 (10.8%)	1638 (19.0%)	4293 (14.3%)
2021	997 (9.8%)	1150 (10.2%)	981 (11.4%)	3128 (10.4%)
2022	1630 (16.0%)	2077 (18.5%)	1428 (16.6%)	5135 (17.1%)
2023	2413 (23.7%)	2557 (22.7%)	1030 (12.0%)	6000 (20.0%)
2024	1477 (14.5%)	2984 (26.5%)	793 (9.2%)	5254 (17.5%)
Health institution				
A	2041 (20.1%)	1784 (15.9%)	1122 (13.0%)	4947 (16.5%)
B	5375 (52.8%)	6263 (55.7%)	4301 (49.9%)	15 939 (53.1%)
C	1316 (12.9%)	916 (8.1%)	798 (9.3%)	3030 (10.1%)
D	518 (5.1%)	998 (8.9%)	897 (10.4%)	2413 (8.0%)
E	877 (8.6%)	1234 (11.0%)	1469 (17.0%)	3580 (11.9%)
F	47 (0.5%)	54 (0.5%)	30 (0.3%)	131 (0.4%)
Medications				
Beta blockers	2769 (27.2%)	4486 (39.9%)	3177 (36.9%)	10 432 (34.7%)
ARB	5400 (53.1%)	6805 (60.5%)	4938 (57.3%)	17 143 (57.1%)
CCB	3010 (29.6%)	3559 (31.6%)	2799 (32.5%)	9368 (31.2%)
Diuretics	4622 (45.4%)	5670 (50.4%)	4223 (49.0%)	14 515 (48.3%)
Lipid	6417 (63.1%)	8078 (71.8%)	5953 (69.1%)	20 448 (68.1%)
Corticosteroids	2097 (20.6%)	2004 (17.8%)	1665 (19.3%)	5766 (19.2%)
NSAIDs	3075 (30.2%)	2460 (21.9%)	2151 (25.0%)	7686 (25.6%)
Antidepressants	2803 (27.6%)	2453 (21.8%)	1999 (23.2%)	7255 (24.2%)
Antipsychotics	757 (7.4%)	817 (7.3%)	774 (9.0%)	2348 (7.8%)
Antidementia drugs	52 (0.5%)	94 (0.8%)	152 (1.8%)	298 (1.0%)
Sulfonylureas	1531 (15.0%)	2260 (20.1%)	2366 (27.5%)	6157 (20.5%)
Thiazolidinediones	231 (2.3%)	379 (3.4%)	312 (3.6%)	922 (3.1%)
Meglitinides	154 (1.5%)	223 (2.0%)	318 (3.7%)	695 (2.3%)
α glucosidase inhibitors	15 (0.1%)	26 (0.2%)	27 (0.3%)	68 (0.2%)
Insulin	3057 (30.0%)	3282 (29.2%)	2923 (33.9%)	9262 (30.8%)
Metformin	6145 (60.4%)	6692 (59.5%)	5485 (63.7%)	18 322 (61.0%)
Anticoagulants	613 (6.0%)	1316 (11.7%)	795 (9.2%)	2724 (9.1%)
Antiplatelet agents	1905 (18.7%)	3034 (27.0%)	2452 (28.5%)	7391 (24.6%)
Proton pump inhibitors	2551 (25.1%)	2883 (25.6%)	2422 (28.1%)	7856 (26.2%)
Hormone replacement therapy	157 (1.5%)	90 (0.8%)	81 (0.9%)	328 (1.1%)
Opioids	2541 (25.0%)	2915 (25.9%)	2569 (29.8%)	8025 (26.7%)
MRA	443 (4.4%)	1009 (9.0%)	316 (3.7%)	1768 (5.9%)
Digoxin	41 (0.4%)	136 (1.2%)	84 (1.0%)	261 (0.9%)
Other anti‐obesity drug	693 (6.8%)	487 (4.3%)	306 (3.6%)	1486 (4.9%)
ARNIs	52 (0.5%)	503 (4.5%)	58 (0.7%)	613 (2.0%)
Laboratory and vital values				
Mean (SD) body mass index	37.6 (48.7)	32.7 (7.25)	33.7 (98.2)	34.7 (60.0)
Mean (SD) systolic blood pressure, mm Hg	132 (16.6)	131 (17.5)	132 (18.1)	132 (17.4)
Mean (SD) diastolic blood pressure, mm Hg	79.0 (10.5)	76.8 (10.7)	76.2 (10.8)	77.4 (10.7)
T2D complications				
Nephropathy	834 (8.2%)	1632 (14.5%)	1007 (11.7%)	3473 (11.6%)
Retinopathy	471 (4.6%)	555 (4.9%)	305 (3.5%)	1331 (4.4%)
Neuropathy	832 (8.2%)	1027 (9.1%)	697 (8.1%)	2556 (8.5%)
Peripheral vascular disease	274 (2.7%)	518 (4.6%)	286 (3.3%)	1078 (3.6%)
Other specified complications	3864 (38.0%)	4074 (36.2%)	2914 (33.8%)	10 852 (36.1%)
Unspecified complications	734 (7.2%)	621 (5.5%)	445 (5.2%)	1800 (6.0%)
Cardiac‐related pre‐existing chronic conditions				
Coronary atherosclerosis and other heart disease	935 (9.2%)	1918 (17.1%)	1151 (13.4%)	4004 (13.3%)
Cerebrovascular disease	149 (1.5%)	235 (2.1%)	241 (2.8%)	625 (2.1%)
Heart failure	557 (5.5%)	1694 (15.1%)	639 (7.4%)	2890 (9.6%)
Peripheral and visceral vascular disease	411 (4.0%)	675 (6.0%)	483 (5.6%)	1569 (5.2%)
Occlusion or stenosis of precerebral or cerebral arteries without infarction	110 (1.1%)	225 (2.0%)	180 (2.1%)	515 (1.7%)
Aortic peripheral and visceral artery aneurysms	73 (0.7%)	114 (1.0%)	100 (1.2%)	287 (1.0%)
Cardiac and circulatory congenital anomalies	69 (0.7%)	113 (1.0%)	80 (0.9%)	262 (0.9%)
Cardiomyopathy	290 (2.9%)	1084 (9.6%)	337 (3.9%)	1711 (5.7%)
Arrhythmias	939 (9.2%)	1971 (17.5%)	1288 (14.9%)	4198 (14.0%)
Pulmonary heart disease	150 (1.5%)	467 (4.2%)	187 (2.2%)	804 (2.7%)
Chronic kidney disease	819 (8.1%)	1716 (15.3%)	1217 (14.1%)	3752 (12.5%)
Hypertension	6110 (60.1%)	7173 (63.8%)	5287 (61.4%)	18 570 (61.8%)

In calibrated analyses, compared with DPP4is, GLP‐1RAs were associated with significantly lower risks of non‐fatal stroke (IRR, 0.73; 95% CI, 0.57–0.93), hospitalisation for UA (IRR, 0.70; 95% CI, 0.50–0.99), 3‐point MACE (IRR, 0.78; 95% CI, 0.62–0.98) and 4‐point MACE (IRR, 0.78; 95% CI, 0.63–0.98). SGLT2is vs. DPP4is showed similar reductions: non‐fatal MI (IRR, 0.75; 95% CI, 0.61–0.91), non‐fatal stroke (IRR, 0.61; 95% CI, 0.50–0.75), hospitalisation for UA (IRR, 0.66; 95% CI, 0.50–0.88), cardiovascular death (IRR, 0.56; 95% CI, 0.41–0.77), 3‐point MACE (IRR, 0.68; 95% CI, 0.57–0.81) and 4‐point MACE (IRR, 0.68; 95% CI, 0.57–0.81). Effectiveness was comparable between GLP‐1RAs and SGLT2is across all outcomes (Figure 1).

**FIGURE 1:**
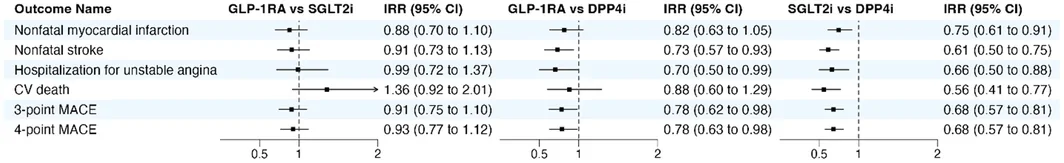
Comparative effectiveness of GLP‐1 receptor agonists (GLP‐1RAs), SGLT2 Inhibitors (SGLT2is) and DPP4 inhibitors (DPP4is) on cardiovascular outcomes. Incident rate ratios (IRRs) and 95% CIs were estimated using propensity score‐matched target trial emulation with negative control outcome calibration. Values < 1 indicate lower risk in the first drug class compared with the comparator.

## 4 Conclusion

In this TTE of adults with T2D, initiation of GLP‐1RAs or SGLT2is was associated with reduced cardiovascular risk compared with DPP4is, with similar effectiveness between GLP‐1RAs and SGLT2is. These findings align with previous evidence [3, 4] and strengthen confidence in the cardiovascular benefits of GLP‐1RAs and SGLT2is in real‐world practice. This study extends this evidence by providing head‐to‐head comparisons across all three drug classes in a broader real‐world population and by applying NCO calibration to evaluate and reduce residual systematic bias.

Clinically, the findings support preferential consideration of GLP‐1RAs or SGLT2is over DPP4is when cardiovascular risk reduction is an important treatment objective. The similar cardiovascular effectiveness of GLP‐1RAs and SGLT2is suggests that selection between these classes may also be guided by patient characteristics, comorbidities, tolerability and treatment preferences.

Limitations include those inherent to EHR‐based observational studies, including potential misclassification, incomplete capture of care outside the health system, missing key clinical variables and residual confounding despite propensity‐score matching and NCO calibration. Cardiovascular outcomes were identified using diagnosis codes and cardiovascular death may be particularly susceptible to misclassification of cause of death. HbA1c, eGFR, albuminuria and diabetes duration could not be reliably incorporated because of substantial missingness or limited historical data, although diagnosis‐based kidney disease and T2D complications were included as proxies for disease severity. Noncardiovascular death was not modelled as a competing event, which may affect the interpretation of cardiovascular outcome estimates. The study was also limited to a single health system with relatively short follow‐up. Finally, the intention‐to‐treat analysis estimates the effect of treatment initiation, regardless of subsequent switching, discontinuation or adherence and therefore should not be interpreted as the effect of sustained treatment.

In summary, this empirically calibrated TTE strengthens the cardiovascular benefits of GLP‐1RAs and SGLT2is over DPP4is and provides reliable comparative evidence to guide treatment selection in routine clinical practice.

## Author Contributions

Authorship has been determined according to ICMJE recommendations.

## Funding

This work was supported in part by National Institutes of Health (U01TR003709, RF1AG077820, R01AG073435, R56AG074604, R01DK128237).

## Disclosure

The funding institutions had no role in the design and conduct of the study; collection, management, analysis, and interpretation of the data; preparation, review, or approval of the manuscript; and decision to submit the manuscript for publication.

## Conflicts of Interest

Dr. David A. Asch is a partner and part‐owner of VAL Health and serves on the advisory boards of Thrive Global and Select. Dr. Yong Chen is a consultant for Pfizer, Johnson & Johnson and Merck, outside the submitted work. The remaining authors declare no conflicts of interest.

## Supporting information


**Table S1:** Specification and emulation of the target trial.
**Table S2:** Variables used in the study evaluating comparative effectiveness.
**Table S3:** List of negative control outcomes.
**Table S4:** Baseline characteristics of patients initiating GLP‐1 receptor agonists versus SGLT2 inhibitors after propensity‐score matching.
**Table S5:** Baseline characteristics of patients initiating GLP‐1 receptor agonists versus DPP4 inhibitors after propensity‐score matching.
**Table S6:** Baseline characteristics of patients initiating SGLT2 inhibitors versus DPP4 inhibitors after propensity‐score matching.
**Table S7:** Event numbers, total follow‐up time and incident rates per 1000 person‐years before propensity‐score matching.
**Table S8:** Event numbers, total follow‐up time and incident rates per 1000 person‐years after propensity‐score matching.
**Figure S1:** Selection of participants.
**Figure S2:** Standard mean difference (SMD) balance after propensity score matching.
**Figure S3:** Distribution of negative control outcomes.
**Figure S4:** Comparative effectiveness of GLP‐1 receptor agonists (GLP‐1RAs), SGLT2 inhibitors (SGLT2is) and DPP4 inhibitors (DPP4is) on cardiovascular outcomes using per‐protocol analysis.
**Figure S5:** Comparative effectiveness of GLP‐1 receptor agonists (GLP‐1RAs), SGLT2 inhibitors (SGLT2is) and DPP4 inhibitors (DPP4is) on cardiovascular outcomes excluding tirzepatide.
**Figure S6:** Comparative effectiveness of GLP‐1 receptor agonists (GLP‐1RAs), SGLT2 inhibitors (SGLT2is) and DPP4 inhibitors (DPP4is) on cardiovascular outcomes using 3‐year risk difference.
**Figure S7:** Comparative effectiveness of GLP‐1 receptor agonists (GLP‐1RAs), SGLT2 inhibitors (SGLT2is) and DPP4 inhibitors (DPP4is) on cardiovascular outcomes in patients aged < 55.
**Figure S8:** Comparative effectiveness of GLP‐1 receptor agonists (GLP‐1RAs), SGLT2 inhibitors (SGLT2is) and DPP4 inhibitors (DPP4is) on cardiovascular outcomes in patients aged 55 to 70.
**Figure S9:** Comparative effectiveness of GLP‐1 receptor agonists (GLP‐1RAs), SGLT2 inhibitors (SGLT2is) and DPP4 inhibitors (DPP4is) on cardiovascular outcomes in patients aged ≥ 70.
**Figure S10:** Comparative effectiveness of GLP‐1 receptor agonists (GLP‐1RAs), SGLT2 inhibitors (SGLT2is) and DPP4 inhibitors (DPP4is) on cardiovascular outcomes in female patients.
**Figure S11:** Comparative effectiveness of GLP‐1 receptor agonists (GLP‐1RAs), SGLT2 inhibitors (SGLT2is) and DPP4 inhibitors (DPP4is) on cardiovascular outcomes in male patients.
**Figure S12:** Comparative effectiveness of GLP‐1 receptor agonists (GLP‐1RAs), SGLT2 inhibitors (SGLT2is) and DPP4 inhibitors (DPP4is) on cardiovascular outcomes in Non‐Hispanic White patients.
**Figure S13:** Comparative effectiveness of GLP‐1 receptor agonists (GLP‐1RAs), SGLT2 inhibitors (SGLT2is) and DPP4 inhibitors (DPP4is) on cardiovascular outcomes in Non‐Hispanic Black patients.
**Figure S14:** Comparative effectiveness of GLP‐1 receptor agonists (GLP‐1RAs), SGLT2 inhibitors (SGLT2is) and DPP4 inhibitors (DPP4is) on cardiovascular outcomes in Hispanic patients.
**Figure S15:** Comparative effectiveness of GLP‐1 receptor agonists (GLP‐1RAs), SGLT2 inhibitors (SGLT2is) and DPP4 inhibitors (DPP4is) on cardiovascular outcomes in Asian patients.

## Data Availability

The results reported in this study are based on detailed individual‐level patient data. The datasets generated and analysed are not publicly available due to the University of Pennsylvania's privacy and confidentiality requirements.
