# Supplementary material for: Comparative Effectiveness of GLP‐1RAs, SGLT2is and DPP4is for Cardiovascular Outcomes in Type 2 Diabetes: An Empirically Calibrated Target Trial Emulation Study
Source: Diabetes Obes Metab. 2026 Aug 27;28(12):11971–7. doi: 10.1111/dom.71244 (PMC13731002; doi:10.1111/dom.71244)
Supplement: Supplementary file 1 [file DOM-28-11971-s001.docx]

Supplementary Appendix to:

**Comparative Effectiveness of GLP-1RAs, SGLT2is, and DPP4is for New-Onset Cardiovascular Outcomes in Type 2 Diabetes: A Debiased Target Trial Emulation Study**

Table S1. Specification and emulation of the target trial.

Table S2. Variables used in the study evaluating comparative effectiveness.

Table S3. List of negative control outcomes.

Table S4. Baseline characteristics of patients initiating GLP-1 receptor agonists versus SGLT2 inhibitors after propensity-score matching.

Table S5. Baseline characteristics of patients initiating GLP-1 receptor agonists versus DPP4 inhibitors after propensity-score matching.

Table S6. Baseline characteristics of patients initiating SGLT2 inhibitors versus DPP4 inhibitors after propensity-score matching.

Table S7. Event numbers, total follow-up time, and incident rates per 1,000 person-years before propensity-score matching.

Table S8. Event numbers, total follow-up time, and incident rates per 1,000 person-years after propensity-score matching.

Figure S1. Selection of participants.

Figure S2. Standard mean difference (SMD) balance after propensity score matching.

Figure S3. Distribution of negative control outcomes.

Figure S4. Comparative effectiveness of GLP-1 receptor agonists (GLP-1RAs), SGLT2 inhibitors (SGLT2is), and DPP4 inhibitors (DPP4is) on cardiovascular outcomes using per-protocol analysis.

Figure S5. Comparative effectiveness of GLP-1 receptor agonists (GLP-1RAs), SGLT2 inhibitors (SGLT2is), and DPP4 inhibitors (DPP4is) on cardiovascular outcomes excluding tirzepatide.

Figure S6. Comparative effectiveness of GLP-1 receptor agonists (GLP-1RAs), SGLT2 inhibitors (SGLT2is), and DPP4 inhibitors (DPP4is) on cardiovascular outcomes using 3-year risk difference.

Figure S7. Comparative effectiveness of GLP-1 receptor agonists (GLP-1RAs), SGLT2 inhibitors (SGLT2is), and DPP4 inhibitors (DPP4is) on cardiovascular outcomes in patients aged <55.

Figure S8. Comparative effectiveness of GLP-1 receptor agonists (GLP-1RAs), SGLT2 inhibitors (SGLT2is), and DPP4 inhibitors (DPP4is) on cardiovascular outcomes in patients aged 55 to 70.

Figure S9. Comparative effectiveness of GLP-1 receptor agonists (GLP-1RAs), SGLT2 inhibitors (SGLT2is), and DPP4 inhibitors (DPP4is) on cardiovascular outcomes in patients aged ≥70.

Figure S10. Comparative effectiveness of GLP-1 receptor agonists (GLP-1RAs), SGLT2 inhibitors (SGLT2is), and DPP4 inhibitors (DPP4is) on cardiovascular outcomes in female patients.

Figure S11. Comparative effectiveness of GLP-1 receptor agonists (GLP-1RAs), SGLT2 inhibitors (SGLT2is), and DPP4 inhibitors (DPP4is) on cardiovascular outcomes in male patients.

Figure S12. Comparative effectiveness of GLP-1 receptor agonists (GLP-1RAs), SGLT2 inhibitors (SGLT2is), and DPP4 inhibitors (DPP4is) on cardiovascular outcomes in Non-Hispanic White patients.

Figure S13. Comparative effectiveness of GLP-1 receptor agonists (GLP-1RAs), SGLT2 inhibitors (SGLT2is), and DPP4 inhibitors (DPP4is) on cardiovascular outcomes in Non-Hispanic Black patients.

Figure S14. Comparative effectiveness of GLP-1 receptor agonists (GLP-1RAs), SGLT2 inhibitors (SGLT2is), and DPP4 inhibitors (DPP4is) on cardiovascular outcomes in Hispanic patients.

Figure S15. Comparative effectiveness of GLP-1 receptor agonists (GLP-1RAs), SGLT2 inhibitors (SGLT2is), and DPP4 inhibitors (DPP4is) on cardiovascular outcomes in Asian patients.

Table S1. Specification and emulation of the target trial.

| Protocol elements | Target trial | Emulation with EHR data |
| --- | --- | --- |
| Eligibility criteria | 1. Adults with type 2 diabetes  2. Age ≥18  3. No diagnosis of type 1 diabetes, end-stage renal disease or dialysis  4. No prior use of study drugs  5. No contraindication to study drugs  6. No prior outcomes of interest | Same as target trial |
| Treatment strategies | Initiation treatment with GLP-1RAs, SGLT2is, or DPP4is | Same as target trial |
| Treatment assignment | Randomization, no blinding | Propensity score matching on the following variables: demographics, comorbidities, body mass index, blood pressure, socioeconomic measures, and healthcare utilization |
| Outcomes | 1. nonfatal myocardial infarction  2. nonfatal stroke  3. CV death  4. hospitalization for unstable angina  5. 3-point MACE (nonfatal MI, nonfatal stroke, CV death)  6. 4-point MACE (nonfatal MI, nonfatal stroke, CV death, hospitalization for UA) | Same as target trial |
| Causal estimand | Intention-to-treat effect | Same as target trial |
| Start and end of follow-up | Follow-up began on the day of treatment initiation, i.e., the index date, and ended at occurrence of the outcome event, death, or the end of the study period | Same as target trial |
| Statistical analysis | Modified Poisson regression model | Same as target trial |

GLP-1RAs, glucagon-like peptide-1 receptor agonists; SGLT2is, sodium-glucose cotransporter-2 inhibitors; DPP4is, dipeptidyl peptidase-4 inhibitors

Table S2. Variables used in the study evaluating comparative effectiveness.

| Variable | Functional form | Values | Detail | Codes/references |
| --- | --- | --- | --- | --- |
| Treatment (i.e., Exposure) |  |  |  |  |
| GLP-1RAs | Indicator | Yes/No | Based on the medication order domain, including albiglutide, dulaglutide, exenatide, liraglutide, lixisenatide, semaglutide, and tirzepatide. | ATC code: A10BJ for selective GLP-1RAs; A10BX16 for tirzepatide |
| SGLT2is | Indicator | Yes/No | Based on the medication order domain, including canagliflozin, dapagliflozin, empagliflozin, ertugliflozin, sotagliflozin, and bexagliflozin. | ATC code: A10BK |
| DPP4is | Indicator | Yes/No | Based on the medication order domain, including sitagliptin, saxagliptin, alogliptin, and linagliptin. | ATC code: A10BH |
| Outcome |  |  |  |  |
| Nonfatal myocardial infarction | Indicator | Yes/No | Based on the diagnosis domain. | ICD code: I21.x, I22.x, I25.2 |
| Nonfatal stroke | Indicator | Yes/No | Based on the diagnosis domain. | ICD code: I61.x, I63.x, I64 |
| CV death | Indicator | Yes/No | Based on the diagnosis and death domains. We used two definitions: 1. Primary ICD codes for CV death; 2. CV events + death document within 30 days. | ICD code only: I46.1, R96.x  ICD code + death document within 30 days: I21.x, I22.x, I61.x, I63.x, I64, I49.x, I44.x, I45.x, I50.1, I50.2, I24.0, I24.8, I20.x, I71.0, I71.x, I80.1-I80.3, I46.0, R57.x |
| Hospitalization for unstable angina | Indicator | Yes/No | Based on the diagnosis and visit domains. Define as ICD code + inpatient/ED setting within [-1, 1] days. | ICD code: I20.x, I24.0, I24.8, I24.9 |
| 3-point MACE | Indicator | Yes/No | Any occurrence of nonfatal MI, nonfatal stroke, or CV death. | NA |
| 4-point MACE | Indicator | Yes/No | Any occurrence of nonfatal MI, nonfatal stroke, CV death, or hospitalization for unstable angina. | NA |
| Confounding variables |  |  |  |  |
| Age (years) | Linear | NA | Based on records in the person domain. | Age is defined as the integer of (index date – birth date)/365.25 |
| Sex | Indicator | Male/Female | Based on records in the person domain. | NA |
| Race/Ethnicity | 6 categories | NHW  NHB  Hispanic  AAPI  Multiple  Other/unknown | Based on records in the person domain. | NA |
| Charlson Comorbidity Index (CCI) score | 2 categories | <3  ≥3 | Based on the visit and diagnosis domains. | NA |
| Pre-existing chronic conditions | Indicator | Yes/No | Based on the visit and diagnosis domains. | <https://github.com/Penncil/Distributional-diagnosis-and-calibration/blob/master/cluster_master.csv> |
| Body Mass Index | Linear | NA | Based on the lab domain. | NA |
| Systolic Blood pressure | Linear | NA | Based on the lab domain. | NA |
| Diastolic Blood Pressure | Linear | NA | Based on the lab domain. | NA |
| Insurance | 4 categories | Commercial  Medicaid  Medicare  Self-Pay/Unknown | Based on the insurance domain. | NA |
| Household median income | 3 categories | <$50,000  $50,000-$100,000  ≥$100,000 | Derived using zip code information. | NA |
| Number of outpatient visits | 2 categories | <10  ≥10 | Based on the visit domain. | NA |
| Number of inpatient visits | 2 categories | 0  ≥1 | Based on the visit domain. | NA |
| Number of ED visits | 2 categories | 0  ≥1 | Based on the visit domain. | NA |
| Year of cohort entry | 6 categories | 2019  2020  2021  2022  2023  2024 | Based on the visit domain. | NA |
| Institution Index | 6 categories | A  B  C  D  E  F | NA | NA |
| Medications | Indicator | Yes/No | Based on the medication domain, including beta blockers, ARB, CCB, diuretics, lipid, corticosteroids, NSAIDs, antidepressants, antipsychotics, antidementia drugs, sulfonylureas, thiazolidinediones, meglitinides, α glucosidase inhibitors, insulin, metformin, anticoagulants, antiplatelet agents, proton pump inhibitors, hormone replacement therapy, opioids, MRA, digoxin, other anti-obesity drug, ARNIs | NA |
| T2D complications | Indicator | Yes/No | Based on diagnosis domain, including nephropathy, retinopathy, neuropathy peripheral vascular disease, other specified complications, unspecified complications | NA |
| Eligibility criteria |  |  |  |  |
| Type 2 diabetes | Indicator | Yes/No | Based on diagnosis domain. | ICD code: E11 |
| Type 1 diabetes | Indicator | Yes/No | Based on diagnosis domain. | ICD code: E10 |
| End stage renal disease/dialysis | Indicator | Yes/No | Based on diagnosis domain. | ICD code: N18.5, N18.6, R88.0, T82.4, T85.611, T85.621, T85.631, T85.71, T76.1, Y84.1, Z48.22, Z49, Z94.0, Z99.2 |

Table S3. List of negative control outcomes.

| Abnormal posture | Inadequate sleep hygiene |
| --- | --- |
| Abnormal pupil | Ingrowing nail |
| Abrasion and/or friction burn of multiple sites | Injury of knee |
| Abrasion and/or friction burn of trunk without infection | Jellyfish poisoning |
| Absence of breast | Kwashiorkor |
| Absent kidney | Lagophthalmos |
| Acquired hallux valgus | Late effect of contusion |
| Acquired keratoderma | Late effect of motor vehicle accident |
| Anal and rectal polyp | Lipid storage disease |
| Anomaly of jaw size | Lymphangioma |
| Benign paroxysmal positional vertigo | Macular drusen |
| Bizarre personal appearance | Malingering |
| Burn of forearm | Marfan’s syndrome |
| Calcaneal spur | Mechanical complication of internal orthopedic device, implant AND/OR graft |
| Cannabis abuse | Melena |
| Changes in skin texture | Minimal cognitive impairment |
| Chondromalacia of patella | Noise effects on inner ear |
| Cocaine abuse | Non-toxic multinodular goiter |
| Colostomy present | Nonspecific tuberculin test reaction |
| Complication due to Crohn’s disease | Opioid intoxication |
| Complication of gastrostomy | Passing flatus |
| Contact dermatitis | Physiological development failure |
| Contusion of knee | Poisoning by tranquilizer |
| Crohn’s disease | Postviral fatigue syndrome |
| Derangement of knee | Presbyopia |
| Developmental delay | Psychalgia |
| Deviated nasal septum | Ptotic breast |
| Difficulty sleeping | Regular astigmatism |
| Disproportion of reconstructed breast | Senile hyperkeratosis |
| Effects of hunger | Social exclusion |
| Endometriosis | Somatic dysfunction of lumbar region |
| Epidermoid cyst | Splinter of face without major open wound |
| Exhaustion due to excessive exertion | Sprain of ankle |
| Feces contents abnormal | Strain of rotator cuff capsule |
| Foreign body in ear | Symbolic dysfunction |
| Foreign body in orifice | Tear film insufficiency |
| Foreskin deficient | Tobacco dependence syndrome |
| Galactosemia | Tooth loss |
| Ganglion cyst | Toxic effect of lead compound |
| Hammer toe | Toxic effect of tobacco and nicotine |
| Hereditary thrombophilia | Tracheostomy present |
| High risk sexual behavior | Unsatisfactory tooth restoration |
| Homocystinuria | Verruca vulgaris |
| Impacted cerumen | Wrist joint pain |
| Impingement syndrome of shoulder region | Wristdrop |

Table S4. Baseline characteristics of patients initiating GLP-1 receptor agonists versus SGLT2 inhibitors after propensity-score matching.

|  | GLP-1RA (N=7,223) | SGLT2i (N=7,223) | SMD |
| --- | --- | --- | --- |
| Age |  |  |  |
| Years; mean (SD) | 60.1 (11.9) | 60.7 (11.7) | -0.05 |
| Sex |  |  |  |
| Female | 3641 (50.4%) | 3509 (48.6%) | 0.04 |
| Male | 3582 (49.6%) | 3714 (51.4%) | -0.04 |
| Race and ethnicity |  |  |  |
| Non-Hispanic White | 3609 (50.0%) | 3669 (50.8%) | -0.02 |
| Non-Hispanic Black | 2432 (33.7%) | 2319 (32.1%) | 0.03 |
| Hispanic | 387 (5.4%) | 397 (5.5%) | -0.006 |
| Asian | 284 (3.9%) | 290 (4.0%) | -0.005 |
| Other/Unknown | 511 (7.1%) | 548 (7.6%) | -0.02 |
| CCI score |  |  |  |
| <3 | 2143 (29.7%) | 2051 (28.4%) | 0.03 |
| ≥3 | 5080 (70.3%) | 5172 (71.6%) | -0.03 |
| Insurance |  |  |  |
| Commercial | 3136 (43.4%) | 3185 (44.1%) | -0.01 |
| Medicaid | 825 (11.4%) | 753 (10.4%) | 0.03 |
| Medicare | 2461 (34.1%) | 2446 (33.9%) | 0.005 |
| Self-Pay/Other | 801 (11.1%) | 839 (11.6%) | -0.02 |
| Zip code-level median household income |  |  |  |
| <$50,000 | 1875 (26.0%) | 1756 (24.3%) | 0.04 |
| $50,000-$100,000 | 3462 (47.9%) | 3527 (48.8%) | -0.02 |
| ≥$100,000 | 1886 (26.1%) | 1940 (26.9%) | -0.02 |
| Number of inpatient visits |  |  |  |
| 0 | 6468 (89.5%) | 6448 (89.3%) | 0.009 |
| ≥1 | 755 (10.5%) | 775 (10.7%) | -0.009 |
| Number of outpatient visits |  |  |  |
| <10 | 1998 (27.7%) | 2039 (28.2%) | -0.01 |
| ≥10 | 5225 (72.3%) | 5184 (71.8%) | 0.01 |
| Number of ED visits |  |  |  |
| 0 | 6421 (88.9%) | 6471 (89.6%) | -0.02 |
| ≥1 | 802 (11.1%) | 752 (10.4%) | 0.02 |
| Health institution |  |  |  |
| A | 1331 (18.4%) | 1264 (17.5%) | 0.02 |
| B | 3901 (54.0%) | 3924 (54.3%) | -0.006 |
| C | 771 (10.7%) | 734 (10.2%) | 0.02 |
| D | 459 (6.4%) | 482 (6.7%) | -0.01 |
| E | 727 (10.1%) | 785 (10.9%) | -0.03 |
| F | 34 (0.5%) | 34 (0.5%) | 0.00 |
| Medications |  |  |  |
| Beta blockers | 2207 (30.6%) | 2267 (31.4%) | -0.02 |
| ARB | 4046 (56.0%) | 4108 (56.9%) | -0.02 |
| CCB | 2194 (30.4%) | 2179 (30.2%) | 0.005 |
| Diuretics | 3340 (46.2%) | 3347 (46.3%) | -0.002 |
| Lipid | 4868 (67.4%) | 4994 (69.1%) | -0.04 |
| Corticosteroids | 1380 (19.1%) | 1308 (18.1%) | 0.02 |
| NSAIDs | 1910 (26.4%) | 1825 (25.3%) | 0.03 |
| Antidepressants | 1813 (25.1%) | 1735 (24.0%) | 0.02 |
| Antipsychotics | 509 (7.0%) | 516 (7.1%) | -0.004 |
| Sulfonylureas | 1275 (17.7%) | 1321 (18.3%) | -0.02 |
| Thiazolidinediones | 199 (2.8%) | 208 (2.9%) | -0.008 |
| Meglitinides | 140 (1.9%) | 126 (1.7%) | 0.02 |
| Insulin | 2071 (28.7%) | 2045 (28.3%) | 0.008 |
| Metformin | 4417 (61.2%) | 4457 (61.7%) | -0.01 |
| Anticoagulants | 495 (6.9%) | 541 (7.5%) | -0.03 |
| Antiplatelet agents | 1511 (20.9%) | 1564 (21.7%) | -0.02 |
| Proton pump inhibitors | 1811 (25.1%) | 1790 (24.8%) | 0.007 |
| Hormone replacement therapy | 84 (1.2%) | 79 (1.1%) | 0.006 |
| Opioids | 1728 (23.9%) | 1744 (24.1%) | -0.005 |
| MRA | 349 (4.8%) | 368 (5.1%) | -0.01 |
| Other anti-obesity drug | 419 (5.8%) | 377 (5.2%) | 0.02 |
| ARNIs | 52 (0.7%) | 62 (0.9%) | -0.02 |
| Laboratory and vital values |  |  |  |
| Mean (SD) body mass index | 36.1 (57.5) | 34.5 (7.52) | 0.03 |
| Mean (SD) systolic blood pressure, mm Hg | 132 (16.8) | 131 (16.9) | 0.02 |
| Mean (SD) diastolic blood pressure, mm Hg | 78.4 (10.4) | 78.1 (10.3) | 0.03 |
| T2D complications |  |  |  |
| Nephropathy | 699 (9.7%) | 740 (10.2%) | -0.02 |
| Retinopathy | 367 (5.1%) | 363 (5.0%) | 0.003 |
| Neuropathy | 618 (8.6%) | 659 (9.1%) | -0.02 |
| Peripheral vascular disease | 217 (3.0%) | 237 (3.3%) | -0.02 |
| Other specified complications | 2731 (37.8%) | 2769 (38.3%) | -0.01 |
| Unspecified complications | 481 (6.7%) | 457 (6.3%) | 0.01 |
| Cardiac-related pre-existing chronic conditions |  |  |  |
| Coronary atherosclerosis and other heart disease | 805 (11.1%) | 852 (11.8%) | -0.02 |
| Cerebrovascular disease | 112 (1.6%) | 113 (1.6%) | -0.001 |
| Heart failure | 493 (6.8%) | 519 (7.2%) | -0.02 |
| Peripheral and visceral vascular disease | 323 (4.5%) | 307 (4.3%) | 0.01 |
| Occlusion or stenosis of precerebral or cerebral arteries without infarction | 99 (1.4%) | 104 (1.4%) | -0.007 |
| Cardiomyopathy | 261 (3.6%) | 279 (3.9%) | -0.01 |
| Arrhythmias | 764 (10.6%) | 809 (11.2%) | -0.02 |
| Pulmonary heart disease | 131 (1.8%) | 141 (2.0%) | -0.01 |
| Chronic kidney disease | 699 (9.7%) | 768 (10.6%) | -0.04 |
| Hypertension | 4392 (60.8%) | 4425 (61.3%) | -0.009 |

Table S5. Baseline characteristics of patients initiating GLP-1 receptor agonists versus DPP4 inhibitors after propensity-score matching.

|  | GLP-1RA (N=5,934) | DPP4i (N=5,934) | SMD |
| --- | --- | --- | --- |
| Age |  |  |  |
| Years; mean (SD) | 62.2 (12.3) | 61.2 (11.9) | -0.08 |
| Sex |  |  |  |
| Female | 3053 (51.4%) | 3160 (53.3%) | 0.04 |
| Male | 2881 (48.6%) | 2774 (46.7%) | -0.04 |
| Race and ethnicity |  |  |  |
| Non-Hispanic White | 3089 (52.1%) | 3021 (50.9%) | -0.02 |
| Non-Hispanic Black | 1747 (29.4%) | 1909 (32.2%) | 0.06 |
| Hispanic | 344 (5.8%) | 318 (5.4%) | -0.02 |
| Asian | 287 (4.8%) | 266 (4.5%) | -0.02 |
| Other/Unknown | 467 (7.9%) | 420 (7.1%) | -0.03 |
| CCI score |  |  |  |
| <3 | 1818 (30.6%) | 1829 (30.8%) | 0.004 |
| ≥3 | 4116 (69.4%) | 4105 (69.2%) | -0.004 |
| Insurance |  |  |  |
| Commercial | 2251 (37.9%) | 2391 (40.3%) | 0.05 |
| Medicaid | 596 (10.0%) | 649 (10.9%) | 0.03 |
| Medicare | 2420 (40.8%) | 2278 (38.4%) | -0.05 |
| Self-Pay/Other | 667 (11.2%) | 616 (10.4%) | -0.03 |
| Zip code-level median household income |  |  |  |
| <$50,000 | 1292 (21.8%) | 1441 (24.3%) | 0.05 |
| $50,000-$100,000 | 3169 (53.4%) | 3012 (50.8%) | -0.05 |
| ≥$100,000 | 1473 (24.8%) | 1481 (25.0%) | 0.003 |
| Number of inpatient visits |  |  |  |
| 0 | 5139 (86.6%) | 5179 (87.3%) | 0.02 |
| ≥1 | 795 (13.4%) | 755 (12.7%) | -0.02 |
| Number of outpatient visits |  |  |  |
| <10 | 1891 (31.9%) | 1778 (30.0%) | -0.04 |
| ≥10 | 4043 (68.1%) | 4156 (70.0%) | 0.04 |
| Number of ED visits |  |  |  |
| 0 | 5283 (89.0%) | 5264 (88.7%) | -0.01 |
| ≥1 | 651 (11.0%) | 670 (11.3%) | 0.01 |
| Health institution |  |  |  |
| A | 889 (15.0%) | 1004 (16.9%) | 0.05 |
| B | 3185 (53.7%) | 3141 (52.9%) | -0.01 |
| C | 640 (10.8%) | 654 (11.0%) | 0.007 |
| D | 462 (7.8%) | 419 (7.1%) | -0.03 |
| E | 738 (12.4%) | 690 (11.6%) | -0.03 |
| F | 20 (0.3%) | 26 (0.4%) | 0.01 |
| Medications |  |  |  |
| Beta blockers | 1882 (31.7%) | 1857 (31.3%) | -0.009 |
| ARB | 3326 (56.0%) | 3268 (55.1%) | -0.02 |
| CCB | 1781 (30.0%) | 1798 (30.3%) | 0.006 |
| Diuretics | 2767 (46.6%) | 2749 (46.3%) | -0.006 |
| Lipid | 3976 (67.0%) | 3946 (66.5%) | -0.01 |
| Corticosteroids | 1140 (19.2%) | 1154 (19.4%) | 0.006 |
| NSAIDs | 1558 (26.3%) | 1619 (27.3%) | 0.02 |
| Antidepressants | 1461 (24.6%) | 1492 (25.1%) | 0.01 |
| Antipsychotics | 471 (7.9%) | 474 (8.0%) | 0.002 |
| Antidementia drugs | 61 (1.0%) | 47 (0.8%) | -0.03 |
| Sulfonylureas | 1295 (21.8%) | 1215 (20.5%) | -0.04 |
| Thiazolidinediones | 198 (3.3%) | 169 (2.8%) | -0.03 |
| Meglitinides | 137 (2.3%) | 126 (2.1%) | -0.02 |
| Insulin | 1762 (29.7%) | 1816 (30.6%) | 0.02 |
| Metformin | 3747 (63.1%) | 3729 (62.8%) | -0.006 |
| Anticoagulants | 453 (7.6%) | 404 (6.8%) | -0.03 |
| Antiplatelet agents | 1388 (23.4%) | 1345 (22.7%) | -0.02 |
| Proton pump inhibitors | 1576 (26.6%) | 1521 (25.6%) | -0.02 |
| Hormone replacement therapy | 61 (1.0%) | 69 (1.2%) | 0.01 |
| Opioids | 1585 (26.7%) | 1536 (25.9%) | -0.02 |
| MRA | 228 (3.8%) | 226 (3.8%) | -0.002 |
| Other anti-obesity drug | 255 (4.3%) | 303 (5.1%) | 0.03 |
| Laboratory and vital values |  |  |  |
| Mean (SD) body mass index | 35.2 (106) | 35.7 (7.94) | 0.01 |
| Mean (SD) systolic blood pressure, mm Hg | 132 (17.2) | 132 (16.8) | -0.004 |
| Mean (SD) diastolic blood pressure, mm Hg | 77.7 (10.5) | 78.0 (10.4) | 0.03 |
| T2D complications |  |  |  |
| Nephropathy | 522 (8.8%) | 537 (9.0%) | 0.009 |
| Retinopathy | 226 (3.8%) | 253 (4.3%) | 0.02 |
| Neuropathy | 459 (7.7%) | 481 (8.1%) | 0.01 |
| Peripheral vascular disease | 170 (2.9%) | 177 (3.0%) | 0.007 |
| Other specified complications | 2086 (35.2%) | 2168 (36.5%) | 0.03 |
| Unspecified complications | 344 (5.8%) | 383 (6.5%) | 0.03 |
| Cardiac-related pre-existing chronic conditions |  |  |  |
| Coronary atherosclerosis and other heart disease | 643 (10.8%) | 630 (10.6%) | -0.008 |
| Cerebrovascular disease | 112 (1.9%) | 100 (1.7%) | -0.02 |
| Heart failure | 359 (6.0%) | 355 (6.0%) | -0.003 |
| Peripheral and visceral vascular disease | 270 (4.6%) | 257 (4.3%) | -0.01 |
| Occlusion or stenosis of precerebral or cerebral arteries without infarction | 87 (1.5%) | 82 (1.4%) | -0.008 |
| Cardiomyopathy | 205 (3.5%) | 183 (3.1%) | -0.02 |
| Arrhythmias | 686 (11.6%) | 626 (10.5%) | -0.03 |
| Pulmonary heart disease | 101 (1.7%) | 91 (1.5%) | -0.01 |
| Chronic kidney disease | 603 (10.2%) | 572 (9.6%) | -0.02 |
| Hypertension | 3485 (58.7%) | 3510 (59.2%) | 0.009 |

Table S6. Baseline characteristics of patients initiating SGLT2 inhibitors versus DPP4 inhibitors after propensity-score matching.

|  | SGLT2i (N=7,505) | DPP4i (N=7,505) | SMD |
| --- | --- | --- | --- |
| Age |  |  |  |
| Years; mean (SD) | 65.0 (12.9) | 64.5 (12.2) | -0.04 |
| Sex |  |  |  |
| Female | 3530 (47.0%) | 3337 (44.5%) | -0.05 |
| Male | 3975 (53.0%) | 4168 (55.5%) | 0.05 |
| Race and ethnicity |  |  |  |
| Non-Hispanic White | 3945 (52.6%) | 3944 (52.6%) | -0.0003 |
| Non-Hispanic Black | 1950 (26.0%) | 1986 (26.5%) | 0.01 |
| Hispanic | 442 (5.9%) | 443 (5.9%) | 0.0006 |
| Asian | 520 (6.9%) | 470 (6.3%) | -0.03 |
| Other/Unknown | 648 (8.6%) | 662 (8.8%) | 0.007 |
| CCI score |  |  |  |
| <3 | 2330 (31.0%) | 2223 (29.6%) | -0.03 |
| ≥3 | 5175 (69.0%) | 5282 (70.4%) | 0.03 |
| Insurance |  |  |  |
| Commercial | 2465 (32.8%) | 2542 (33.9%) | 0.02 |
| Medicaid | 626 (8.3%) | 619 (8.2%) | -0.003 |
| Medicare | 3461 (46.1%) | 3246 (43.3%) | -0.06 |
| Self-Pay/Other | 953 (12.7%) | 1098 (14.6%) | 0.05 |
| Zip code-level median household income |  |  |  |
| <$50,000 | 1414 (18.8%) | 1456 (19.4%) | 0.01 |
| $50,000-$100,000 | 4182 (55.7%) | 4012 (53.5%) | 0.04 |
| ≥$100,000 | 1909 (25.4%) | 2037 (27.1%) | -0.05 |
| Number of inpatient visits |  |  |  |
| 0 | 6464 (86.1%) | 6516 (86.8%) | 0.02 |
| ≥1 | 1041 (13.9%) | 989 (13.2%) | -0.02 |
| Number of outpatient visits |  |  |  |
| <10 | 2588 (34.5%) | 2527 (33.7%) | -0.02 |
| ≥10 | 4917 (65.5%) | 4978 (66.3%) | 0.02 |
| Number of ED visits |  |  |  |
| 0 | 6754 (90.0%) | 6778 (90.3%) | 0.01 |
| ≥1 | 751 (10.0%) | 727 (9.7%) | -0.01 |
| Health institution |  |  |  |
| A | 1014 (13.5%) | 1113 (14.8%) | 0.04 |
| B | 3943 (52.5%) | 4045 (53.9%) | 0.03 |
| C | 692 (9.2%) | 641 (8.5%) | -0.02 |
| D | 760 (10.1%) | 715 (9.5%) | -0.02 |
| E | 1069 (14.2%) | 955 (12.7%) | -0.05 |
| F | 27 (0.4%) | 36 (0.5%) | 0.02 |
| Medications |  |  |  |
| Beta blockers | 2658 (35.4%) | 2683 (35.7%) | 0.007 |
| ARB | 4318 (57.5%) | 4351 (58.0%) | 0.009 |
| CCB | 2345 (31.2%) | 2346 (31.3%) | 0.0003 |
| Diuretics | 3557 (47.4%) | 3549 (47.3%) | -0.002 |
| Lipid | 5204 (69.3%) | 5237 (69.8%) | 0.01 |
| Corticosteroids | 1341 (17.9%) | 1324 (17.6%) | -0.006 |
| NSAIDs | 1726 (23.0%) | 1705 (22.7%) | 0.003 |
| Antidepressants | 1648 (22.0%) | 1657 (22.1%) | -0.01 |
| Antipsychotics | 561 (7.5%) | 540 (7.2%) | -0.01 |
| Antidementia drugs | 91 (1.2%) | 78 (1.0%) | -0.02 |
| Sulfonylureas | 1914 (25.5%) | 1727 (23.0%) | -0.06 |
| Thiazolidinediones | 279 (3.7%) | 276 (3.7%) | -0.002 |
| Meglitinides | 206 (2.7%) | 188 (2.5%) | -0.02 |
| Insulin | 2171 (28.9%) | 2150 (28.6%) | -0.006 |
| Metformin | 4766 (63.5%) | 4663 (62.1%) | -0.03 |
| Anticoagulants | 677 (9.0%) | 726 (9.7%) | 0.02 |
| Antiplatelet agents | 1994 (26.6%) | 1932 (25.7%) | -0.02 |
| Proton pump inhibitors | 1965 (26.2%) | 1937 (25.8%) | -0.009 |
| Opioids | 1963 (26.2%) | 1945 (25.9%) | -0.005 |
| MRA | 304 (4.1%) | 384 (5.1%) | 0.04 |
| Digoxin | 73 (1.0%) | 67 (0.9%) | -0.007 |
| Other anti-obesity drug | 278 (3.7%) | 292 (3.9%) | 0.009 |
| ARNIs | 58 (0.8%) | 100 (1.3%) | 0.03 |
| Laboratory and vital values |  |  |  |
| Mean (SD) body mass index | 33.1 (69.8) | 32.4 (7.07) | -0.097 |
| Mean (SD) systolic blood pressure, mm Hg | 132 (17.6) | 132 (17.5) | -0.02 |
| Mean (SD) diastolic blood pressure, mm Hg | 76.7 (10.7) | 76.8 (10.3) | 0.01 |
| T2D complications |  |  |  |
| Nephropathy | 840 (11.2%) | 919 (12.2%) | 0.03 |
| Retinopathy | 278 (3.7%) | 329 (4.4%) | 0.03 |
| Neuropathy | 589 (7.8%) | 619 (8.2%) | 0.01 |
| Peripheral vascular disease | 261 (3.5%) | 276 (3.7%) | 0.01 |
| Other specified complications | 2512 (33.5%) | 2659 (35.4%) | 0.04 |
| Unspecified complications | 397 (5.3%) | 379 (5.1%) | -0.01 |
| Cardiac-related pre-existing chronic conditions |  |  |  |
| Coronary atherosclerosis and other heart disease | 990 (13.2%) | 1059 (14.1%) | 0.02 |
| Cerebrovascular disease | 163 (2.2%) | 146 (1.9%) | -0.02 |
| Heart failure | 581 (7.7%) | 680 (9.1%) | 0.04 |
| Peripheral and visceral vascular disease | 399 (5.3%) | 391 (5.2%) | -0.004 |
| Occlusion or stenosis of precerebral or cerebral arteries without infarction | 155 (2.1%) | 145 (1.9%) | -0.01 |
| Cardiomyopathy | 318 (4.2%) | 400 (5.3%) | 0.04 |
| Arrhythmias | 1055 (14.1%) | 1064 (14.2%) | 0.003 |
| Pulmonary heart disease | 161 (2.1%) | 184 (2.5%) | 0.02 |
| Chronic kidney disease | 999 (13.3%) | 995 (13.3%) | -0.001 |
| Hypertension | 4497 (59.9%) | 4544 (60.5%) | 0.01 |

Table S7. Event numbers, total follow-up time, and incident rates per 1,000 person-years before propensity-score matching.

|  | GLP-1RA (N=10,174) | SGLT2i (N=11,249) | DPP4i (N=8,617) |
| --- | --- | --- | --- |
| **Follow-up** |  |  |  |
| Total follow-up time – no. of person-year | 26,696 | 22,944 | 25,991 |
| Median [Q1, Q3] | 2.26 [1.18, 4.28] | 1.64 [0.69, 3.14] | 2.95 [1.51, 4.63] |
| **Number of events** |  |  |  |
| Nonfatal myocardial infarction | 345 | 504 | 453 |
| Nonfatal stroke | 398 | 455 | 547 |
| Hospitalization for unstable angina | 129 | 162 | 199 |
| CV death | 92 | 117 | 181 |
| 3-point MACE | 732 | 954 | 996 |
| 4-point MACE | 790 | 1,017 | 1,069 |
| All-cause mortality | 259 | 354 | 654 |
| **Incidence rate per 1,000 person-year** |  |  |  |
| Nonfatal myocardial infarction | 12.9 | 22.0 | 17.4 |
| Nonfatal stroke | 14.9 | 19.8 | 21.0 |
| Hospitalization for unstable angina | 4.8 | 7.1 | 7.7 |
| CV death | 3.4 | 5.1 | 7.0 |
| 3-point MACE | 27.4 | 41.6 | 38.3 |
| 4-point MACE | 29.6 | 44.3 | 41.1 |
| All-cause mortality | 9.7 | 15.4 | 25.2 |

Table S8. Event numbers, total follow-up time, and incident rates per 1,000 person-years after propensity-score matching.

|  | GLP-1RA vs SGLT2i | | GLP-1RA vs DPP4i | | SGLT2i vs DPP4i | |
| --- | --- | --- | --- | --- | --- | --- |
|  | GLP-1RA | SGLT2i | GLP-1RA | DPP4i | SGLT2i | DPP4i |
| **Follow-up** |  |  |  |  |  |  |
| Total follow-up time – no. of person-year | 18,683 | 16,132 | 15,701 | 18,547 | 15,913 | 22,909 |
| Median [Q1, Q3] | 2.20 [1.16, 4.21] | 1.89 [0.82, 3.50] | 2.31 [1.20, 4.26] | 3.20 [1.63, 4.73] | 1.72 [0.73, 3.30] | 3.05 [1.55, 4.67] |
| **Number of events** |  |  |  |  |  |  |
| Nonfatal myocardial infarction | 280 | 260 | 243 | 266 | 313 | 382 |
| Nonfatal stroke | 301 | 271 | 261 | 320 | 304 | 452 |
| Hospitalization for unstable angina | 103 | 85 | 82 | 104 | 108 | 149 |
| CV death | 78 | 519 | 69 | 70 | 73 | 119 |
| 3-point MACE | 576 | 549 | 495 | 565 | 608 | 814 |
| 4-point MACE | 622 | 47 | 530 | 603 | 648 | 868 |
| All-cause mortality | 205 | 157 | 187 | 264 | 251 | 437 |
| **Incidence rate per 1,000 person-year** |  |  |  |  |  |  |
| Nonfatal myocardial infarction | 15.0 | 16.1 | 15.5 | 14.3 | 19.7 | 16.7 |
| Nonfatal stroke | 16.1 | 16.8 | 16.6 | 17.3 | 19.1 | 19.7 |
| Hospitalization for unstable angina | 5.5 | 5.3 | 5.2 | 5.6 | 6.8 | 6.5 |
| CV death | 4.2 | 2.9 | 4.4 | 3.8 | 4.6 | 5.2 |
| 3-point MACE | 30.8 | 32.2 | 31.5 | 30.4 | 38.2 | 35.5 |
| 4-point MACE | 33.3 | 34.0 | 33.8 | 32.5 | 40.7 | 37.9 |
| All-cause mortality | 10.1 | 9.7 | 11.9 | 14.2 | 15.7 | 19.1 |

Figure S1. Flow diagram of patient inclusion and exclusion criteria.

Figure S2. Covariate balance before and after propensity score matching. Standardized mean differences (SMDs) for baseline covariates before (red) and after (blue) 1:1 propensity score matching are shown for the three pairwise treatment comparisons: GLP-1RA vs SGLT2i, GLP-1RA vs DPP4i, and SGLT2i vs DPP4i. For each comparison, the upper section displays the 20 covariates with the largest absolute SMDs before matching, and the lower section displays the 20 covariates with the largest absolute SMDs after matching. Vertical dashed lines indicate the absolute SMD threshold of 0.1.

Figure S3. Distribution of negative control outcomes (NCOs). Each blue point represents the effect estimate and standard error for one distinct NCO analyzed separately using the same study design and analytic approach as the corresponding primary outcome. The distribution of negative control estimates was used to characterize systematic error and calibrate the primary results for each pairwise treatment comparison. The vertical black line indicates an incidence rate ratio of 1.0. The grey area marked by the dashed line indicates estimates with a traditional p-value of <0.05. The orange line represents a calibrated p-value of <0.05 using NCOs. The expected absolute systematic error is shown in each panel.

For each pairwise treatment comparison, the estimated log incidence rate ratios for the negative control outcomes were used to estimate an empirical null distribution. For each negative control outcome, we repeated the PS estimation and outcome modeling to obtain log IRR estimates $\hat{\beta}_{m}$ with standard errors $\hat{\sigma}_{m}$. We assume the true systematic bias from the $m$-th negative control, denoted $\eta_{m}$, follows a normal distribution:

$$\eta_{m}\sim N\left( \mu, \tau^{2} \right), \hat{\beta}_{m}\sim N\left( \eta_{m}, \hat{\sigma}_{m}^{2} \right),$$

where $\mu$ and $\tau^{2}$ characterize the empirical null distribution of systematic bias. Parameters $\mu$ and $\tau^{2}$ can be estimated via maximum likelihood, and primary outcome estimates $\hat{\beta}_{0}$ with variance $\hat{\sigma}_{0}^{2}$ were then calibrated as:

$${\tilde{\beta}_{0}=\hat{\beta}}_{0}-\hat{\mu}, \tilde{\sigma}_{0}^{2}=\hat{\sigma}_{0}^{2}+\hat{\tau}^{2}.$$

Further details on the empirical calibration procedure are provided in Schuemie et al and Wang et al.^1–3^

References:

1.	Schuemie MJ, Ryan PB, DuMouchel W, et al. Interpreting observational studies: why empirical calibration is needed to correct p‐values. *Stat Med* 2014; 33: 209–218.

2.	Schuemie MJ, Hripcsak G, Ryan PB, et al. Empirical confidence interval calibration for population-level effect estimation studies in observational healthcare data. *Proceedings of the National Academy of Sciences* 2018; 115: 2571–2577.

3.	Wang H, Zhang B, Lei Y, et al. Distributional Diagnosis and Calibration with Negative Controls for Outcome-wide Real-world Evidence. Epub ahead of print 13 July 2026. DOI: 10.64898/2026.07.08.26357550.

Figure S4. Comparative effectiveness of GLP-1 receptor agonists (GLP-1RAs), SGLT2 inhibitors (SGLT2is), and DPP4 inhibitors (DPP4is) on cardiovascular outcomes using per-protocol analysis.

Figure S5. Comparative effectiveness of GLP-1 receptor agonists (GLP-1RAs), SGLT2 inhibitors (SGLT2is), and DPP4 inhibitors (DPP4is) on cardiovascular outcomes excluding tirzepatide.

Figure S6. Comparative effectiveness of GLP-1 receptor agonists (GLP-1RAs), SGLT2 inhibitors (SGLT2is), and DPP4 inhibitors (DPP4is) on cardiovascular outcomes using 3-year risk difference.

Figure S7. Comparative effectiveness of GLP-1 receptor agonists (GLP-1RAs), SGLT2 inhibitors (SGLT2is), and DPP4 inhibitors (DPP4is) on cardiovascular outcomes in patients aged <55.

Figure S8. Comparative effectiveness of GLP-1 receptor agonists (GLP-1RAs), SGLT2 inhibitors (SGLT2is), and DPP4 inhibitors (DPP4is) on cardiovascular outcomes in patients aged 55 to 70.

Figure S9. Comparative effectiveness of GLP-1 receptor agonists (GLP-1RAs), SGLT2 inhibitors (SGLT2is), and DPP4 inhibitors (DPP4is) on cardiovascular outcomes in patients aged ≥70.

Figure S10. Comparative effectiveness of GLP-1 receptor agonists (GLP-1RAs), SGLT2 inhibitors (SGLT2is), and DPP4 inhibitors (DPP4is) on cardiovascular outcomes in female patients.

Figure S11. Comparative effectiveness of GLP-1 receptor agonists (GLP-1RAs), SGLT2 inhibitors (SGLT2is), and DPP4 inhibitors (DPP4is) on cardiovascular outcomes in male patients.

Figure S12. Comparative effectiveness of GLP-1 receptor agonists (GLP-1RAs), SGLT2 inhibitors (SGLT2is), and DPP4 inhibitors (DPP4is) on cardiovascular outcomes in Non-Hispanic White patients.

Figure S13. Comparative effectiveness of GLP-1 receptor agonists (GLP-1RAs), SGLT2 inhibitors (SGLT2is), and DPP4 inhibitors (DPP4is) on cardiovascular outcomes in Non-Hispanic Black patients.

Figure S14. Comparative effectiveness of GLP-1 receptor agonists (GLP-1RAs), SGLT2 inhibitors (SGLT2is), and DPP4 inhibitors (DPP4is) on cardiovascular outcomes in Hispanic patients.

Figure S15. Comparative effectiveness of GLP-1 receptor agonists (GLP-1RAs), SGLT2 inhibitors (SGLT2is), and DPP4 inhibitors (DPP4is) on cardiovascular outcomes in Asian patients.
